# VvARF19 represses VvLBD13-mediated cell wall degradation to delay softening of grape berries

**DOI:** 10.1093/hr/uhae322

**Published:** 2024-11-18

**Authors:** Meng Li, Changjiang Nie, Shanshan He, Zhirui Xue, Jiajun Li, Zhiqian Li, Chang He, Xianbo Zheng, Bin Tan, Jun Cheng, Wei Wang, Jidong Li, Xia Ye, Jiancan Feng

**Affiliations:** College of Horticulture, Henan Agricultural University, 218 Pingan Road, Zhengzhou 450046, China; College of Horticulture, Henan Agricultural University, 218 Pingan Road, Zhengzhou 450046, China; College of Horticulture, Henan Agricultural University, 218 Pingan Road, Zhengzhou 450046, China; College of Horticulture, Henan Agricultural University, 218 Pingan Road, Zhengzhou 450046, China; College of Horticulture, Henan Agricultural University, 218 Pingan Road, Zhengzhou 450046, China; College of Horticulture, Henan Agricultural University, 218 Pingan Road, Zhengzhou 450046, China; International Joint Laboratory of Henan Horticultural Crop Biology, 218 Pingan Road, Zhengzhou 450046, China; College of Horticulture, Henan Agricultural University, 218 Pingan Road, Zhengzhou 450046, China; International Joint Laboratory of Henan Horticultural Crop Biology, 218 Pingan Road, Zhengzhou 450046, China; College of Horticulture, Henan Agricultural University, 218 Pingan Road, Zhengzhou 450046, China; International Joint Laboratory of Henan Horticultural Crop Biology, 218 Pingan Road, Zhengzhou 450046, China; College of Horticulture, Henan Agricultural University, 218 Pingan Road, Zhengzhou 450046, China; International Joint Laboratory of Henan Horticultural Crop Biology, 218 Pingan Road, Zhengzhou 450046, China; College of Horticulture, Henan Agricultural University, 218 Pingan Road, Zhengzhou 450046, China; International Joint Laboratory of Henan Horticultural Crop Biology, 218 Pingan Road, Zhengzhou 450046, China; College of Horticulture, Henan Agricultural University, 218 Pingan Road, Zhengzhou 450046, China; International Joint Laboratory of Henan Horticultural Crop Biology, 218 Pingan Road, Zhengzhou 450046, China; College of Forestry, Henan Agricultural University, 218 Pingan Road, Zhengzhou 450046, China; College of Horticulture, Henan Agricultural University, 218 Pingan Road, Zhengzhou 450046, China; International Joint Laboratory of Henan Horticultural Crop Biology, 218 Pingan Road, Zhengzhou 450046, China; College of Horticulture, Henan Agricultural University, 218 Pingan Road, Zhengzhou 450046, China; International Joint Laboratory of Henan Horticultural Crop Biology, 218 Pingan Road, Zhengzhou 450046, China

## Abstract

Fruit softening directly impacts its storage life, transportability, and customer acceptance. Auxin plays a key role during fruit ripening, but the underlying mechanisms of how auxin regulates fruit softening remain unclear. In this study, we investigated the regulatory roles of auxin on berry cell wall degradation during grape (*Vitis vinifera* L.) softening. During grape berry development, berry firmness and auxin content both firstly increase and then decrease, and peaks occur 4–6 weeks after full blooming. Exogenous NAA (α-naphthalene acetic acid, a synthetic auxin) treatment inhibits berry softening by delaying propectin, cellulose, and hemicellulose degradation, which maintains cell wall integrity in the grape flesh. Weighted gene co-expression network analysis (WGCNA) showed that *VvLBD13*, correlated with VvARF19, could be a key gene in this delaying of berry softening, and is involved in auxin signal transduction and cell wall degradation metabolism. Overexpression and transient overexpression of *VvLBD13* in tomato or in grape berry indicate that *VvLBD13* accelerates hemicellulose degradation by binding the promoters of *VvXTH10* (xyloglucan endotransglucosylase/hydrolase 10) and *VvEXPLA1* (expansion-like A1), which results in rapid softening after veraison. Collectively, this research furnishes an exhaustive understanding of the auxin-driven regulatory mechanisms of grape berry softening.

## Introduction

The softening of grape berries is an important physiological change during the onset of fruit ripening, which is a highly coordinated process. Softening begins prior to the rapid sugar accumulation and color change of the peel [1]. A significant change in programming towards grape softening occurs at veraison [2]. At or just after veraison, fruit firmness quickly decreases, which has a great impact on fruit storage life, transportability, and customer acceptability. Consequently, it is quite necessary to understand the molecular mechanism controlling grape berry softening, including metabolic changes and transcriptional regulation, as a foundation for prolonging shelf-life and improving the market value of grape berries.

Plant hormones work together with other signaling pathways in a complex and sophisticated network to control and coordinate grape berry softening [3, 4]. It is well known that auxin and abscisic acid (ABA) are two key hormones during grape berry ripening [3]. This is especially true for auxin, as the levels of indole-3-acetic acid (IAA) increase rapidly from pollination to fruit set and quickly decrease to low concentrations close to veraison, which implies that auxin plays an important role during the transition from unripe to ripe [5, 6]. Treatment with α-naphthalene acetic acid (NAA) or benzothiazole-2-oxyacetic acid delays grape berry softening during the pre-veraison period, and delays ripening onset by inhibiting degradation of cellulose and homogalacturonan [5, 7], further suggesting that auxin has important roles in the softening of grapes. However, it is not well understood how auxin affects ripening onset and/or grape softening, nor have the key regulatory genes related to grape softening been identified.

Fruit softening is closely related to changes in the cell wall, including its components and structural integrity, during the ripening process [8]. The cell wall is a matrix of polysaccharides, hemicellulose, and cellulose microfibrils built into a cross-lamellar arrangement [8, 9]. During fruit ripening and softening, pectins are depolymerized or degraded by pectin esterase (PME), pectate lyase (PL), and polygalacturonase (PG), and hemicellulose and cellulose are degraded by xyloglucan endotransglucosylase/hydrolase (XTH) and cellulase, respectively [8, 10]. The *SlPL* gene has been verified to play an important part in tomato (*Solanum lycopersicum*) ripening [11], and *SlBES1* and *SlLOB1* are two transcription factors that regulate tomato softening by transcriptional regulation of *SlPMEU1* (related to pectin de-methylesterification) and of *SlEXP1* (encodes a wall-loosening protein), respectively [12, 13]. The softening of strawberry (*Fragaria × ananassa*) is affected by β-galactosidase (β-Gal) [14], PG, or PL activity [15], and silencing of the genes encoding the above key enzymes impedes fruit softening, while overexpression of *FvXTH9* and *FvXTH6* accelerates fruit ripening by the modification of cell wall components [16]. Moreover, *FvWRKY48* binds to the *FvPLA* promoter, regulating pectate lyase gene expression, pectin degradation and fruit softening [17]. Grape berry softening also involves depolymerization of xyloglucans and pectic polysaccharides, resulting in decreasing hemicellulose and pectin content during softening [18]. In comparison, one main reason underlying textural differences between hard-flesh and soft-flesh grapes is the pectin structural integrity during ripening and how much firmness decreases during storage [19]. Transcriptome analysis indicates that pectin, cellulose, and hemicellulose degradation are correlated with fruit firmness [13, 15, 20]. Based on previous reports, grape fruit softening involves degradation of pectin, hemicellulose, and cellulose, but the transcriptional regulation and the key transcription factors affecting berry softening have yet to be discovered.

**Figure 1:**
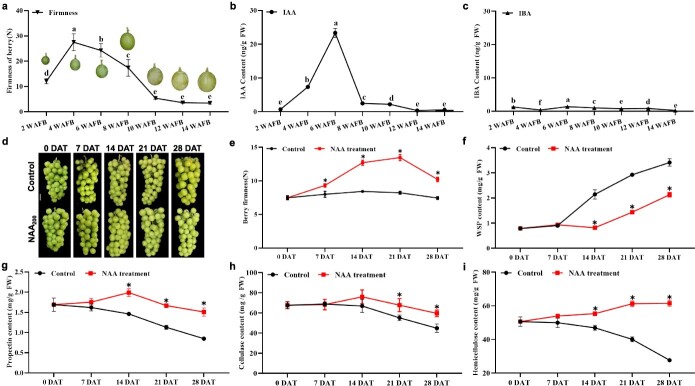
Auxin delayed the berry softening process and inhibited the degradation of cell wall components. **a**–**c** Untreated berries were harvest over 14 WAFB. **a** Firmness during grape ripening and softening. The content of IAA (**b**) and IBA (**c**) during grape ripening and softening. **d**–**i** Clusters were treated with auxin NAA or water at 9 WAFB and then assessed over 28 DAT. **d** Photographs of control and NAA treatment of grape clusters. **e** Changes in fruit firmness. **f** WSP. **g** Propectin. **h** Cellulose. **i** Hemicellulose. Values represent the average ± standard deviation. Different letters and asterisks represent a significant difference (*P* < 0.05) using one-way ANOVA and Student’s *t*-test, respectively.

To understand the roles of physiological changes, transcriptional regulation, and auxin in the softening of grape, as well as the potential regulatory mechanisms initiating cell wall degradation, the auxin content and softening process of ‘Shine Muscat’ grapes (*Vitis labruscana* Bailey × *V. vinifera* L.) were analyzed during berry ripening and softening. Additionally, some berries were sprayed with NAA before veraison. Pectin, hemicellulose, and cellulose contents and their enzyme activities were analyzed, and cell wall microstructure and RNA-seq analysis were performed in response to auxin. Our results indicated that *VvLBD13* could be a key node regulating berry softening, which was correlated with auxin signal transduction and cell wall degradation metabolism.

## Results

### Auxin delayed the berry softening process and inhibited the degradation of cell wall components

Fruit softening is influenced by the degradation of cell wall components. Grape berry firmness quickly increased during 2–4 weeks after full blooming (WAFB), and maintained a higher level during 4–6 WAFB, then rapidly reduced during 8–10 WAFB at veraison (Fig. 1a). IAA content showed a trend similar to that of berry firmness (Supplementary Data Fig. S1a), first increasing and then decreasing, and the maximum IAA content was at 6 WAFB. At 8 WAFB, the IAA content quickly decreased to a lower level, and then grape berries showed accelerated softening (Fig. 1b and c). After veraison (10 WAFB), both firmness and IAA content were maintained at a relative lower level (Fig. 1a and b). Unsurprisingly, the 3-indolebutyric acid (IBA) content maintained a lower level during berry development and ripening (Fig. 1c).

To further understand the roles of auxin in berry softening, grape clusters were treated with NAA at veraison (9 WAFB). The results showed that NAA treatment significantly delayed grape berry ripening and softening (Fig. 1d and e). After NAA treatment, berry firmness first increased during 0–21 days after treatment (DAT) and then decreased, while berry firmness in the control (water treatment) remained steady during 0–28 DAT (Fig. 1e). Compared with the control, firmness in treated berries increased by 42, 62, and 32% at 14, 21, and 28 DAT, respectively. NAA treatment increased the titratable acid (TA) and decreased the soluble solids content (SSC) in berries (Fig. S1b). The water-soluble pectin (WSP) content of both NAA-treated and control berries generally increased over time, although the WSP content was higher in the control than NAA-treated berries during 14–28 DAT, with the largest difference (51%) observed at 14 DAT (Fig. 1f). Propectin content was far higher in NAA-treated berries than in the control, with the largest difference (52%) at 14 DAT (Fig. 1g). During 14–28 DAT, slightly higher cellulose content was observed in NAA-treated berries compared with the control (Fig. 1h). Notably, NAA treatment had a significant influence on the hemicellulose content. The hemicellulose content gradually decreased over time in the control, but remained stable and even slightly increased in response to NAA treatment (Fig. 1h). At 28 DAT, the maximum difference in hemicellulose content between NAA-treated and the control berries was observed, with hemicellulose content 2.22 times higher in NAA-treated berries than in the control (Fig. 1i). The above results strongly indicated that auxin delayed the berry softening process and prevented the degradation of cell wall components.

**Figure 2:**
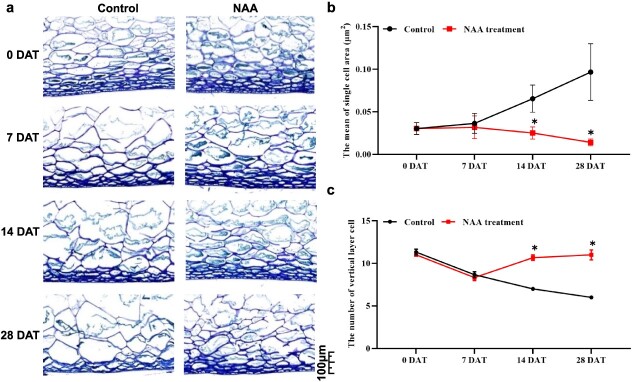
Light microscopy images of grape berry sections in control and NAA-treated groups. **a** Epidermal and pulp microstructure of fresh berries. Scale bar is 100 μm. **b** Single-cell mean area. **c** Number of vertical layer cells. Values are the average ± standard deviation. Asterisks represent a significant difference using Student’s *t*-test (*P* < 0.05).

Typically, the softening of fruit involves the breakdown of cell walls and the above results only indicated that auxin affects grape berry ripening and softening. Whether auxin treatment promotes cell wall microstructure integrity in grape berries remains uncertain. Therefore, we investigated how NAA influenced cell wall integrity at three time points (7, 14 and 28 DAT). The results showed that NAA treatment remained at a level of integrity to control fruit morphology at 28 DAT (Fig. 2a). At veraison (7 DAT), the grape flesh of the control began to lose cell wall integrity compared with NAA treatment, while the cell wall structure of NAA-treated berries maintained good integrity (Fig. 2a and b). During 14–28 DAT, most cell walls in the grape flesh of the control had been broken, and the cells in the epidermal layer were uneven in size and irregularly arranged. However, the cell regularity and cell wall integrity were maintained in the NAA-treated berries, with a greater number of cell layers and a significantly decreased single-cell area during 14–28 DAT, compared with the controls (Fig. 2a–c). Auxin treatment promoted cell wall microstructure integrity in grape berry, potentially elucidating the reason for the change in firmness following auxin treatment.

### Cell wall-degrading enzyme activities during berry softening were inhibited with auxin treatment

The activities of several cell wall metabolism- or breakdown-related enzymes, namely pectinesterase (PE), PG, PL, xyloglucan endotransglycosylase (XET), cellulase (Cx), and β-galactosidase (β-Gal), were detected in grape berries treated with NAA or water. The PG, PE, PL, XET, and Cx activities in both groups gradually increased with time, but NAA treatment decreased enzyme activity in comparison with the control over 28 DAT (Supplementary Data Fig. S2). PE activity after auxin treatment was significantly lower than that of the control by 26–36% (Supplementary Data Fig. S2a). Auxin treatment significantly lowered PG enzyme activity by 87% at 7 DAT and 24% at 21 DAT (Supplementary Data Fig. S2b), and PL activity, with a maximum reduction of 31.5% at 21 DAT (Supplementary Data Fig. S2c). XET activity was significantly lowered over the 28 DAT by 28–55% (Supplementary Data Fig. S2d) and Cx activity was decreased by 27.91 and 37.71% in response to NAA treatment at 21 and 28 DAT, respectively (Supplementary Data Fig. S2e). β-Gal activity was also significantly decreased over 28 DAT, with a maximum reduction of 61% (Supplementary Data Fig. S2f). These results indicated that NAA impeded the escalating activities of cell wall-related enzymes in softening of grape berries.

### Transcriptional analysis of genes related to auxin and cell wall metabolism in NAA-treated grape berries

**Figure 3:**
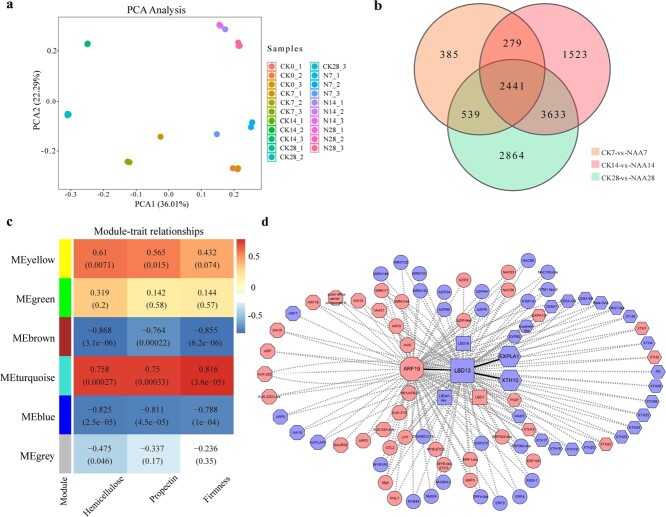
RNA-seq analysis revealed DEGs in response to auxin treatments. Red and blue DEGs stand for upregulated and downregulated genes, respectively. **a** Principal component analysis of 11 664 DEGs. **b** Number of DEGs in the each period shown in a Venn diagram. **c** Module–trait relationships determined by WGCNA. Heat map of correlation between DEGs and hemicellulose content, propectin content, and firmness. Values in the grid represent the correlations between DEGs and hemicellulose, propectin, and firmness. **d** Computed regulatory network of transcription factors and auxin- and cell wall-related pathways. The shapes represent genes within different pathways: octagons, auxin pathway; rectangles, LBD family; hexagons, cell wall-related; circles, other transcription factors.

To more comprehensively evaluate the functions of auxin in the process of fruit softening at the transcriptional level, RNA sequencing was performed using grape berries with NAA and water treatment. The results showed 11 664 differentially expressed genes (DEGs, Supplementary Data Table S2) between NAA treatment and the control. Principal component analysis confirmed that most transcribed genes were significantly altered by exogenous NAA (Fig. 3a). A Venn diagram revealed that 2441 DEGs (Supplementary Data Table S3) were commonly expressed throughout all stages in both the NAA and the control groups (Fig. 3b). These 2441 DEGs were further classified by Gene Ontology (GO) enrichment and Kyoto Encyclopedia of Genes and Genomes (KEGG) enrichment analysis (Supplementary Data Fig. S4a–c), which showed that xyloglucan metabolic process and plant hormonal signaling transduction pathway were significantly enriched. These observations indicated that NAA treatment inhibiting the softening of grape berries mainly influenced plant hormone signaling transduction and xyloglucan metabolism.

To explore the metabolic networks and transcription factors associated with grape berry softening, we employed propectin content, hemicellulose content, and firmness to establish correlations with 2441 DEGs using weighted gene co-expression network analysis (WGCNA). The DEGs were divided into six modules. Each module had a distinct correlation with hemicellulose content, with the coefficient varying from −0.868 to 0.758, propectin content varying from −0.811 to 0.75, and firmness varying from −0.855 to 0.816 (Fig. 3c). The module MEturquoise showed significant positive correlations with hemicellulose content (*R* = 0.758), propectin content (*R* = 0.75), and firmness (*R* = 0.816) (Fig. 3c). This intramodular connectivity indicated that genes within the auxin metabolism pathway (including *VvAUX/IAA*s, *VvSAUR*, *VvARF*s, etc.) and the cell wall degradation pathway (including *VvXTH*s, *VvEXP*s, and *VvCEL*s, etc.) exhibited strong co-expression patterns, which implied their critical roles in auxin-delayed berry softening. *VvLBD13* and *VvARF19* were key node genes that were closely correlated with auxin metabolism and cell wall degradation (Fig. 3d). Auxin response factor (ARF) serves as a vital component in the auxin signaling pathway, facilitating the plant's response to auxin. In auxin metabolism, 16 out of 20 DEGs were significantly induced following NAA treatment. Notably, the expression of *VvARF19* showed a marked increase with NAA treatment, peaking at levels four times higher than the control at 7 DAT (Supplementary Data Fig. S3a). Additionally, *LBD* genes, which encode plant-specific transcription factors, are crucial for the development of lateral organs. Specifically, *VvLBD13* exhibited a reduction in expression at 3, 7, 14, and 28 DAT following NAA treatment (Supplementary Data Fig. S3b). Within the cell wall degradation pathway, 22 of the 28 DEGs were significantly downregulated under NAA treatment. Especially, the expression of *VvXTH10* and *VvEXPLA1* was decreasing during 14 and 28 DAT (Supplementary Data Fig. S3b). In summary, *VvLBD13* may be a key gene in grapes responding to auxin signaling and delaying fruit softening, serving as a critical hub gene connecting auxin signal transduction and cell wall metabolism.

### *VvARF19* correlated with *VvLBD13* is involved in berry development and softening

**Figure 4:**
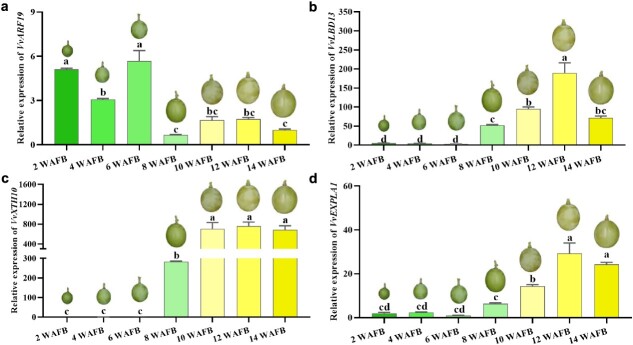
Expression analysis of *VvARF19*, *VvLBD13*, *VvXTH10*, and *VvEXPLA1* during berry development and softening of ‘Shine Muscat’. Values are average ± standard deviation. Different letters represent a significant difference using one-way ANOVA (*P* < 0.05).

To elucidate the roles of the key node genes of *VvARF19* and *VvLBD13* as well as *VvXTH10* and *VvEXPLA1* during grape berry development and softening, their expression patterns were further analyzed during normal berry maturation. *VvARF19* displayed the highest expression levels at 6 WAFB and then expression decreased from 8 to 14 WAFB, which was similar to the trend of IAA content in berries (Fig. 4a). *VvLBD13* gradually increased with berry softening, which displayed a significantly negative correlation (*R* = –0.82) with fruit firmness. Expression patterns of all the *VvXTH*s and *VvEXP*s involved in WGCNA analysis were analyzed during berry developing and ripening, and it was shown that *VvLBD13* displayed a highly correlated expression with *VvXTH10* (*R* = 0.8), which encodes a xyloglucan endotransglucosylase/hydrolase, and *VvEXPLA1* (*R* = 0.90), which encodes a wall-loosening protein (Fig. 4b and c). These gene expression results further implied their potential involvement in grape berry softening. When integrated with existing literature and our experimental findings, we identified a regulatory module composed of *VvARF19*, *VvLDB13*, *VvXTH10*, and *VvEXPLA1*, which could orchestrate the softening process in grape berries.

### VvARF19 is involved in hemicellulose degradation through inhibited VvLBD13 transcription

**Figure 5:**
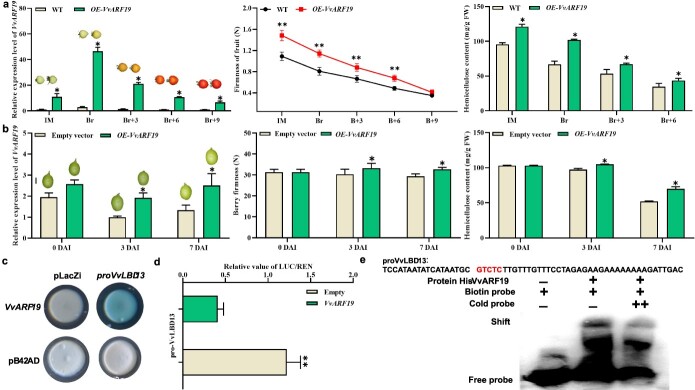
Functional analysis of *VvARF19* and the regulatory relationship of VvARF19 and *VvLBD13*. **a** Relative expression of *VvARF19*, firmness changes, and hemicellulose contents in WT and OE*-VvARF19* tomato fruits. **b** Relative expression of *VvARF19*, firmness changes, and hemicellulose contents in grape berries with transient OE*-VvARF19* and empty vector as control. **c** Y1H assays validated that *VvARF19* could bind to the *VvLBD13* promoter. The empty vector pB42AD was used as negative control. **d** The LUC reporter assay indicated that VvARF19 downregulated *VvLBD13* expression. The reporter pro*VvLBD13*:LUC together with the effector 35S:*VvARF19* was infiltrated into *N. benthamiana* leaves to measure LUC activity. **e** The EMSA showed that VvARF19 directly bound to the *VvLBD13* promoter. ‘–’ means absence, ‘+’ means presence, ‘++’ indicates increasing amounts of cold probe for competition. Values are average ± standard deviation. Asterisks represent significant differences using Student’s *t*-test (*P* < 0.05).

To ascertain whether VvARF19 governs the expression of genes associated with fruit softening, *VvARF19* was stably overexpressed in tomato and transiently overexpressed in grape berries. In transgenic tomato fruits, overexpression of *VvARF19* increased fruit firmness by 16.6–41.3% and hemicellulose content by 25.1–52.5% during fruit ripening (Fig. 5a). Transiently overexpressing *VvARF19* slightly increased berry firmness and inhibited hemicellulose degradation (Fig. 5b). These results showed that VvARF19 acted as a negative regulator of fruit softening and inhibited hemicellulose degradation in the grape berry.

To decipher the regulatory mechanism by which VvARF19 affects fruit softening and hemicellulose degradation, VvARF19 was analyzed for its ability to regulate transcripts of *VvLBD13* using yeast one-hybrid (Y1H) assays (Fig. 5c), and it was shown that VvARF19 could bind to the promoter of *VvLBD13*. The dual-luciferase (LUC) reporter assay further proved that VvARF19 inhibited the promoter activities of *VvLBD13*, and VvARF19 negatively regulated the expression of *VvLBD13* (Fig. 5d). RT–PCR further showed that transiently overexpressing *VvARF19* in grape berries also reduced the *VvLBD13*, *VvXTH10*, and *VvEXPLA1* transcript levels (Supplementary Data Fig. S5a–c). The electrophoretic mobility shift assay (EMSA) subsequently confirmed that VvARF19 bound to the promoter of the *VvLBD13* (Fig. 5e). These results strongly suggested that VvARF19 acted as a negative regulator of grape fruit softening.

### VvLBD13 promoted fruit softening by activating expression of cell wall-related genes

**Figure 6:**
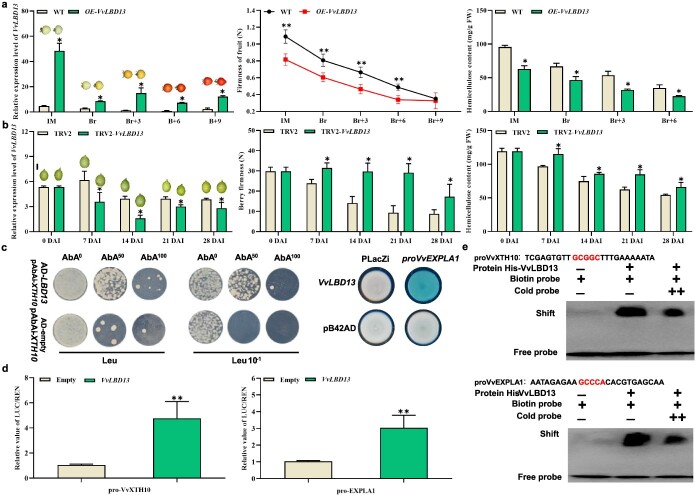
Functional analysis of *VvLBD13* and the regulatory relationship of VvLBD13 on *VvXTH10* and *VvEXPLA1*. **a** Relative expression of *VvLBD13*, firmness, and hemicellulose content in WT and OE*-VvLBD13* tomato fruits. **b** Relative expression of *VvLBD13*, firmness, and hemicellulose content in grape berries infiltrated with TRV2-*VvLBD13* and co-injected with TRV1, with TRV2 and TRV1 co-injection as control. **c** Y1H assay validating that VvLBD13 could bind to the *VvXTH10* and *VvEXPLA1* promoters. The empty vector pB42AD or AD was used as negative control. **d** LUC reporter assay showing that VvLBD13 upregulated *VvXTH10* and *VvEXPLA1* transcript levels. The reporters *proVvXTH10* and *VvEXPLA1*: LUC, together with the effector 35S:*VvLBD13*, were infiltrated into *N. benthamiana* leaves to measure LUC activity. **e** EMSA showed that VvLBD13 could directly bind to the *VvXTH10* and *VvEXPLA1* promoters. ‘–’ means absence, ‘+’ means presence, ‘++’ indicates increasing amounts of cold probe for competition. Values are average ± standard deviation. Asterisks represent significant differences using Student’s *t*-test (*P* < 0.05).

To further investigate the function of VvLBD13 in grape fruit softening, we overexpressed *VvLBD13* in tomato or grape berries (Fig. 6). Overexpression of *VvLBD13* in tomato did not affect fruit color, but the transgenic tomato fruits softened earlier than the wild type (WT) and contained significantly less hemicellulose (Fig. 6a). Both transient overexpression and downregulation of *VvLBD13* in grape berries were also investigated. Compared with grape berries transiently transfected with the empty vector, overexpression of *VvLBD13* slightly decreased berry firmness and hemicellulose content at 3 and 7 days after infiltration (DAI) (Fig. S6a). Downregulation of *VvLBD13* greatly increased berry firmness and inhibited hemicellulose degradation. Berry softening stopped from 7 to 21 DAI, and berry firmness was 3.1 times higher than that in the control at 21 DAI (Fig. 6b).

Based on the WGCNA analysis, we inferred that VvLBD13 may regulate transcription of genes related to cell wall degradation. The YIH assay, LUC reporter assay, and EMSA were employed to reveal that cell wall genes were regulated by VvLBD13 and to validate target binding sites with VvLBD13. The results of Y1H showed that VvLBD13 could bind *VvXTH10* and *VvEXPLA1* promoters (Fig. 6c). Subsequently the LUC assays proved that VvLBD13 bound to the promoters of *VvXTH10* and *VvEXPLA1* and activated the expression of *VvXTH10* and *VvEXPLA1*. In grape berries transiently silenced for *VvLBD13*, the expression levels of two *VvLBD13* target genes, *VvXTH10* and *VvEXPLA1*, were both decreased in fruits (Supplementary Data Fig. S7), but their expression was upregulated in grape berries transiently overexpressing *VvLBD13* (Supplementary Data Fig. S6b). Furthermore, EMSA further confirmed the physical binding of the VvLBD13 recombinant protein to the DNA probes from the *VvXTH10* and *VvEXPLA1* promoter regions (Fig. 6e). In conclusion, VvLBD13 influenced the expression of cell wall-related genes, thereby regulating the softening of grape flesh.

### *VvXTH10* and *VvEXPLA1* were involved in fruit softening

**Figure 7:**
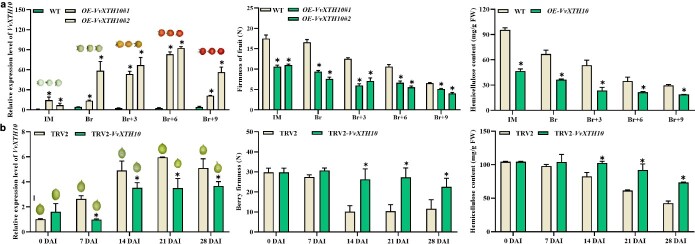
Functional analysis of *VvXTH10*. **a** Relative *VvXTH10* expression, berry firmness, and hemicellulose content in WT and OE-*VvXTH10* tomato fruits. **b** Relative *VvXTH10* expression, berry firmness, and hemicellulose content in grape berries infiltrated in TRV2-*VvXTH10* and co-injected with TRV1; co-injection of TRV2 and TRV1 was the control. Values are average ± standard deviation. Asterisks represent significant differences using Student’s *t*-test (*P* < 0.05).

To identify the possible role of xyloglucan endotransglucosylase/hydrolase in grape fruit softening, *VvXTH10* was stably overexpressed in tomato and transiently overexpressed in grape berries. The results indicated that transgenic tomato fruits overexpressing *VvXTH10* exhibited quite lower fruit firmness and hemicellulose content (Fig. 7a). Transiently overexpressing *VvXTH10* in grape berries greatly decreased berry firmness and hemicellulose content compared with the control (Supplementary Data Fig. S8). In contrast, silencing of *VvXTH10* in grape berries produced the opposite phenotype, with delayed berry softening. Grape berries silenced for *VvXTH10* remained highly firm during 21 DAI (at around 30 N), while berry firmness of the control quickly decreased to 10 N at 14 DAI (Fig. 7b). In addition, the hemicellulose content was significantly lower than that of the control berries. Besides, in transgenic tomato fruits overexpressing *VvEXPLA1*, expression levels of *VvEXPLA1* were consistently four times higher than those in WT tomatoes (Supplementary Data Fig. S9a), and berry firmness in the OE-*VvEXPLA1#1/2* transgenic lines was significantly lower compared with the WT (Supplementary Data Fig. S9b). Additionally, these transgenic lines exhibited lower levels of propectin, while the levels of WSP were significantly elevated (Supplementary Data Fig. S9c and d). These findings suggested that *VvXTH10* and *VvEXPLA1* played a positive role in grape berry softening.

## Discussion

### Auxin negatively regulated grape softening by inhibiting degradation of propectin and hemicellulose

Fruit ripening and softening is a complex series of processes, in which auxin plays a regulatory role. As the peach (*Prunus persica* L.) fruit matures, auxin and ethylene are both involved in softening [21, 22]. Peaches with the stony hard trait do not soften due to lower IAA content in fruits, and only begin to soften when high ethylene production is induced by NAA treatment [22]. On the other hand, in strawberry, auxin is a key negative signal coordinating with ABA to regulate fruit development, ripening, and softening [23]. In grape berries, auxin plays a negative role in ripening and softening [5, 7], with IAA levels rapidly increasing after pollination and remaining until fruit set, after which it quickly decreases to low concentrations close to veraison. Moreover, grape berry ripening is delayed by exogenous auxin treatment during the pre-veraison period [7]. In our study similar results were obtained, and the highest auxin content was observed at 6 WAFB, and rapidly decreased to a quite lower level at 8 WAFB. Concurrently, the berries began to soften during 6–8 WAFB, and then softening accelerated soften during 8–10 WAFB. At the same time, NAA treatment at 9 WAFB could reverse this fast berry softening process, delaying berry softening and the veraison stage. Therefore, we inferred that auxin is a key negative signal during grape berry ripening, and decreasing auxin was a momentous pre-required factor to initiate fruit softening.

The dynamics between cell wall polysaccharides, including pectins, hemicellulose, and cellulose, have been correlated with fruit softening in pear (*Pyrus bretschneideri*) [24], kiwifruit (*Actinidia chinensis*) [25], tomato [11], and Younai plums (*Prunus salicina* Lindl. cv. ‘Younai’) [26]. During the softening of ‘Kyoho’ grape berries, the degradation of pectins and xyloglucan are involved in berry softening [18]. Moreover, auxin treatment of grape berries delays ripening by inhibiting the degradation of homogalacturonan and cellulose and reducing the content of methyl esterified homogalacturonan (meHG) [7]. Our study further verifies that exogenous spraying of developing grape berries with NAA reduced the contents of WSP and delayed the degradation of propectin and cellulose. However, the hemicellulose content remained stable and even slightly increased in the NAA-treated berries, compared with its normal downward trend in the control. It is noteworthy that an irreplaceable role of hemicellulose (main components: xyloglucans) in the cell wall structure, interacting very compactly with cellulose and linking with pectin through rhamnogalacturonan I, constitutes the load-bearing network of plant primary cell walls, thereby strengthening the cell wall [27]. Previous reports have proposed that potential interactions between hemicellulose and pectins impact cell adhesion and cell porosity, which were significantly responsible for tomato fruit firmness [28]. The increasing hemicellulose content in NAA-treated berries further suggested that inhibiting the degradation of hemicellulose may represent a key strategy in delaying the softening of grape berries.

How do the interconnected mechanisms of cell walls facilitate the softening of fruits? During this process, the quantities of at least nine types of cell wall structures or metabolic proteins undergo significant changes, predominantly including PE, PG, PL, XET, Cx, and β-Gal [8, 10, 27]. Auxin precisely modulates the activity of several essential cell wall remodeling enzymes, playing a pivotal role in the orchestration of fruit softening through meticulous control of the cell wall—a mechanism that is prevalent across a variety of fruit cells [29, 30]. In Japanese plums (*P. salicina* L.), auxin expedites fruit softening by regulating the expression of genes associated with pectin [26]. Conversely, in strawberries, treatment with NAA postpones softening primarily by diminishing the activities of Cx, XET, PG, and rhamnogalacturonan IV lyase [31]. In the case of grape berries, NAA treatment not only delays ripening but also suppresses the transcription of pectin-related genes such as *VvPG*, *VvPL*, *VvXTH*, and *VvPME* [7]. Our findings support these observations, showing that NAA treatment significantly reduced the activities of PE, PG, PL, XET, Cx, and β-Gal in ‘Shine Muscat’ grapes. This indicated that NAA inhibited the enzymes responsible for cell wall degradation, thus slowing down the softening of grape berries.

### Auxin maintained the cell wall integrity of grape berries

Fruit softening is manipulated by the secretion of a range of degradation enzymes into the cell wall, which damages the morphology of cell wall structure. Correlation analysis between firmness and histological characteristics indicates that tomato fruit firmness is positively correlated with pericarp tissue cell size [32]. The softening of apple (*Malus domestica*) fruits is accompanied by breakage or deformation of cells in pericarp tissue [33]. In grape berry softening, the number of longitudinal layer cells or single-cell area gradually decreased or increased, respectively [34], which was reversed by NAA treatment in our study. These results showed that NAA treatment could maintain cell wall integrity, delaying the softening of grape berries.

### Auxin signal transduction and xyloglucan metabolism play important roles in NAA-delayed grape berry softening

Auxin regulates fruit ripening and softening. Exogenous auxin represses the ripening of tomato fruit by influencing ripening-related gene expression [35]. In NAA-treated grape berries, transcriptome analysis indicated that most DEGs related to auxin conjugation (GH3-like, Aux/IAAs) are upregulated, and considerable cross-talk of ethylene and auxin is observed [7]. In the present study, genes associated with the auxin signal transduction pathway were upregulated in grape berries by NAA treatment, which further verified that auxin as well as auxin-related genes may have a negative regulatory role in grape berry ripening and softening.

Previous work has reported that the dual roles of xyloglucan endotransglucosylases/hydrolases (XTHs) coordinate newly transported xyloglucan into existing wall-bound xyloglucan chains or restructure hemicellulose by catalyzing the transglucosylation of previously wall-bound xyloglucan molecules [36]. Remarkably, fruit firmness seems to be in direct correlation to the maintenance of xyloglucan contents [37, 38]. In tomato fruit, *SIXTH5* plays an important role in fruit softening, since xyloglucan depolymerization occurs after ripening [39]. In NAA-treated strawberry, genes related to cell wall degradation (*FaEXPA*s, *FaXTH*s, *FaPG*s, etc.) were downregulated at 48 h [31]. Decreased expression of DEGs encoding cell wall catabolic enzymes and increased expression of DEGs encoding cellulose synthases were also observed in grape berries during a 48-h time-course following auxin treatment [7]. These results suggest that the cell wall degradation-related genes that are correlated with fruit ripening and softening respond to NAA treatment. In this study, most DEGs encoding cell wall catabolic enzymes were downregulated in response to NAA treatment; in particular, xyloglucan metabolism was significantly enriched among the auxin-treated DEGs based on GO analyses. Additionally, the majority of *VvXTH*s from xyloglucan metabolism exhibited downregulation in NAA-treated berries, which coincided with higher hemicellulose content and high firmness. Thus, these findings indicated that xyloglucan metabolism plays pivotal roles in grape berry softening.

### The VvARF19–VvLBD13–VvXTH10/VvEXPLA1 module regulated grape berry softening in response to auxin signal

Auxin signal usually modulates the expression of a wide variety of downstream genes, primarily through ARFs [40]. Previous work demonstrated the functions of ARFs in fruit ripening. In tomato, *SlARF2B* and *SlARF2A* primarily function in close association with fruit ripening by enhancing fruit firmness and chlorophyll contents [41, 42]. During papaya (*Carica papaya* L.) ripening, *CpARF2* expression increases by inducing ethylene levels, but *CpARF2* expression is repressed by NAA treatment [40]. During grape berry ripening and softening, low auxin concentrations do not generally promote the expression of ARFs [43]. However, numerous auxin-regulated genes are induced by NAA, including those encoding *VvARF5* and *VvARF18* in ‘Pinot Noir’ grape berries [7]. In the present study, we identified six key *VvARF* genes through transcriptomic and RT–PCR analyses, with the expression of *VvARF19* significantly upregulated under NAA treatment. Our data indicated that overexpression of *VvARF19* in tomato fruit delayed softening via repressing the degradation of hemicellulose. Transient overexpression of *VvARF19* in grape berries was consistent with the above observations. These results implied that *VvARF19* played a key role in the auxin regulation of grape berry softening.

The relationship between ARFs and fruit softening has been highlighted, yet the mechanisms underlying its regulatory role with respect to downstream genes remain poorly understood. In *Arabidopsis thaliana*, *AtLBD16* is a downstream target gene of *AtARF7/19*, which is involved in adventitious root formation [44]. In apple, *MdARF5* stimulates the expression of *MdACS3a* to induce ethylene production and initiate fruit ripening [45]. In our study, the co-expression, Y1H, and dual-regulatory analyses indicated that *VvLBD13* was a target gene of VvARF19. Biochemical experiments established that VvARF19 bound to the AuxRE-like elements (GTCTC) in the promoter of *VvLBD13*, as demonstrated by EMSA. These observations collectively indicated that *VvARF19*, a negative regulator of grape berry softening, participated in the regulation of *VvLBD13* by directly binding to its promoter.

LOB/LBD genes are notable intermediates in regulatory networks defined in other species. For instance, LOBs can regulate AP2, WOX, E2F, or KNOX transcription factors, while binding sites for BZR1, ARF, and NAC transcription factors are located upstream of different LOBs in *A. thaliana* [46]. By analyzing a co-expression network of grape softening-related genes, we identified *VvLBD13* as a potential intermediate factor linking *VvARF19* and *VvXTH10* or *VvEXPLA1* genes. Further experiments showed that berry softening was accelerated in *VvLBD13*-overexpressing tomato fruit and grape berries, which accelerated the disassembly of hemicellulose, whereas the results of downregulation of *VvLBD13* were opposite to those of *VvLBD13* overexpression. Our data present direct evidence that VvLBD13 functions as a positive regulator of grape fruit softening by promoting hemicellulose degradation.

Recently, transcriptional modulation of LBD and cell wall-modifying genes was regarded as an important means of activating or repressing fruit softening during maturation. LOBs can bind the promoter and activate EXP gene expression in *A. thaliana* [47] and *Musa acuminata* [48]. It is noteworthy that *SlLOB1* [13] can activate the *SlPL* gene *in vitro* and has a positive effect on *PL* expression, resulting in rapid fruit softening [11]. In our study, we revealed that VvLBD13 could activate the expression of *VvXTH10* or *VvEXPLA1* by binding to the promoter, as demonstrated by Y1H, LUC, and EMSA assays. Furthermore, overexpression of *VvLBD13* in grape berries promoted expression of the *VvXTH10* and *VvEXPLA1* genes. Similarly, silencing of *VvLBD13* in grape berries also suppressed the expression of the *VvXTH10* and *VvEXPLA1* genes. These findings elucidated a regulatory mechanism wherein VvLBD13–VvXTH10/VvEXPLA1 modulated the softening of grape berries by influencing the degradation of hemicellulose.

### Conclusions

**Figure 8:**
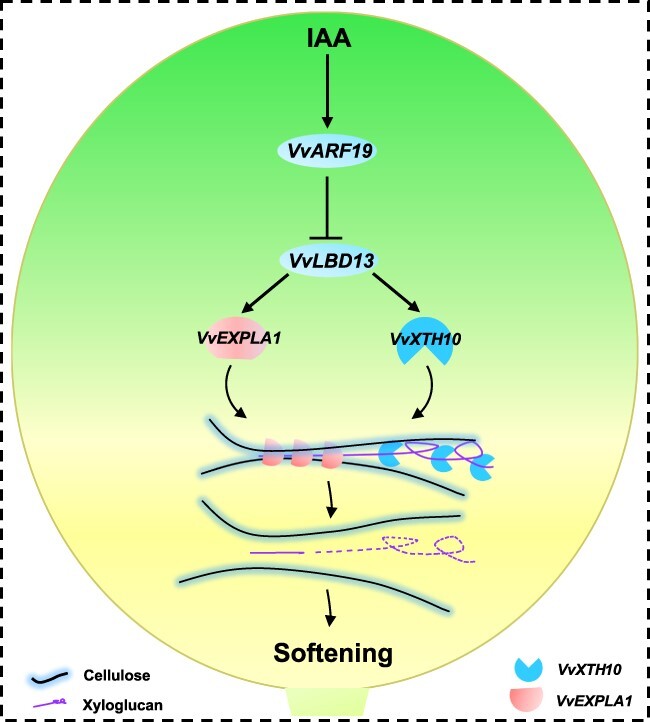
A model of VvARF19-mediated negative regulation of softening in grape berries by suppressing the transcription of *VvLBD13*. Before veraison, a decreasing auxin concentration and lower abundance of *VvARF19* transcripts relieves repression of the transcription of *VvLBD13*. The resulting higher abundance of *VvLBD13* transcripts induces a transcriptional burst of cell wall modification (*VvXTH10* and *VvEXPLA1*) by directly targeting the *VvLBD13*-specific motif within their promoters. Consequently, the activation of *VvXTH10* and *VvEXPLA1* promotes cell wall degradation and contributes to grape berry softening.

In conclusion, we propose a molecular mechanism based on the VvARF19–VvLBD13–VvXTH10/VvEXPLA1 cascade during grape berry softening. As grape berries develop, auxin rapidly decreases to a low level during 6–8 WAFB, leading to low expression of *VvARF19* at 8 WAFB. Before veraison (9 WAFB), *VvLBD13* transcript levels gradually increase as the negative regulation by *VvARF19* is weakened. Increased *VvLBD13* levels then synchronously induce higher expression of *VvXTH10* and *VvEXPLA1*. As a result, *VvXTH10* and *VvEXPLA1* increase hemicellulose hydrolysis in the berry, leading to rapid softening of the grape berry (Fig. 8). Overall, our results suggest the presence of dynamic mechanisms that finely modulate grape berry softening, with the VvARF19–VvLBD13–VvXTH10/VvEXPLA1 cascade governing the degradation of xyloglucan.

## Materials and methods

### Plant material and NAA treatments

Grape vines of the cultivar ‘Shine Muscat’ (*Vitis labruscana* Bailey × *V. vinifera* L.) were maintained at the Henan Agricultural Fruit Experimental Station (Zhengzhou, Henan, China). Grape clusters were collected from 2 to 14 WAFB every 2 weeks, with six to eight grape clusters collected on each sampling date. During the pre-veraison period (9 WAFB, 1 week before veraison), 200 uniform grape clusters were treated with NAA (0.2 g/L) and water as the control [4]. Grape clusters of ‘Shine Muscat’ were sprayed with 200 mg/L NAA solution at 9 WAFB, and grape clusters sprayed with purified water were used as the controls. Two days after treatments, all clusters were bagged with new bags. Around 12 clusters from the control and treated grapes were harvested at 0, 7, 14, 21, and 28 DAT. The grapes were rapidly carried to the laboratory at ambient temperatures.

### Measurement of berry traits and auxin contents

Berry firmness, auxin contents, SSC, and TA were gauged using 20–30 berries from six clusters for each quality parameter, and each experiment was performed at least three times. Berry firmness was analyzed using the TA.XT Plus Texture Analyser (Stable Micro Systems Ltd, Godalming, UK). Berry SSC (Brix) was analyzed by a refractometer (PAL-2, Atago, Japan). To measure TA, 5 g fresh berry was mashed and were titrated with 0.1 N sodium hydroxide. The samples were rapidly frozen in liquid nitrogen for RNA extraction and cell wall content and enzyme activity measurements.

For endogenous auxin measurement, 2 g fresh berry powder from different developing stages was evaluated. Auxins, including IAA and IBA, were measured using the HPLC–MS/MS system referred to by Godoy *et al*. [49]. Three biological replicates were performed for the NAA and control groups of data in each period.

### Histological analysis of grape berry

To observe structure changes of the cell wall in berry in response to NAA treatment, berries from 0, 7, 14, and 21 DAT were transversely cut into 5- to 7-mm slices, and immediately immersed in 50% FAA buffer (main components: 5% glacial acetic acid, 5% formaldehyde, 50% ethanol). After dehydrating by a series of graded ethanols and embedding in paraffin, the samples were divided into 5-μm cross-sections using an ultra-thin semiautomatic microtome (KD-3358). Sections were dyed in 0.5% (w/v) toluidine blue, and then were photographed under a microscope (Zeiss, Oberkochen, Germany). The number of vertical layer cells and single-cell mean area were analyzed with three biological replicates per sampling point.

### RNA extraction and RNA-seq analysis

Total RNA was isolated from 0.2 g of grape berry according to the manufacture’s protocol (Labhelper RH1401, Beijing Labhelper Biotechnology Co. Ltd, China). The concentration of RNA was gauged using a NanoDrop 2000 (Thermo, USA), and 1% agarose gel electrophoresis was carried out to assess the quality of RNA. Twenty-one RNA-seq libraries were constructed from four sampling points (0, 7, 14, and 28 days after NAA treatment) and sequenced using a DNBSEQ-50 platform at the Shenzhen Genomics Institute (BGI, Shenzhen, China). Clean reads (Supplementary Data Table S1) were mapped to the *V. vinifera* reference genome (https://www.ncbi.nlm.nih.gov/data-hub/genome/GCF_000003745.3/) utilizing the Hierarchical Indexing for Spliced Alignment of Transcripts (HISAT, version 2.0.4) software [50]. Under the accession number PRJNA1116830, the original sequencing results have been placed in the NCBI Sequence Read Archive (SRA).

### Measure of cell wall polysaccharide contents and cell wall metabolism-associated enzyme activity

Propectin and WSP were detected by carbazole colorimetry according to Lin *et al*. [51]. Hemicellulose content was determined by combining hydrochloric acid hydrolysis and a dinitrosalicylic acid (DNS) kit (BXW-1-G Suzhou, Jiangsu, China). The cellulose content was measured with a cellulose content assay kit (CLL-1-Y, Suzhou, Jiangsu, China). Kit assays were carried out referring to the manufacturer’s instructions.

The activities of PL, XET, PE, PG, β-Gal, and Cx were analyzed according to previously described methods [51].

### Gene expression analysis

DEGs (Supplementary Data Table S2) between NAA treatments and the control at 0, 7, 14, and 28 DAT were identified, and the default criteria of DEGs among different groups were required: both fold change ≥2.0 and *P*-value <0.05. GO and KEGG enrichment analyses were executed using the goseq R package and the R clusterProfiler package.

The 2441 DEGs (Supplementary Data Table S3) were used with the R package of WGCNA to calculate the correlation coefficients with hemicellulose, propectin, and firmness [52]. Network constructions and module detections of WGCNA were performed with the following parameters: deep split threshold of 0.25, *R*^2^ cut-off of 0.85, ‘signed’ network type, and a minimum module size of 25. The correlation coefficients of six modules were obtained using the automatic network construction and default settings. The hub genes within the module related to the trait of interest were identified by analysis of gene significance and module membership values.

### Dual-luciferase reporter assay

The amplifying full-length coding sequences (CDSs) of *VvLBD13* or *VvARF19* from cDNA or the promoters of *VvXTH10*, *VvEXPLA1*, and *VvLBD13* from genomic DNA by PCR were cloned into plant expression vector 35S:SAK_277_ or pGreenII 0800-LUC, respectively [53]. All the above sequences were derived from ‘Shine Muscat’ and primer sequences are shown in Supplementary Data Table S4. The effector vectors (35S:SAK_277_-*VvLBD13* or *VvARF19* and 35S:SAK_277_ as empty vector) and reporter vectors (pGreenII 0800-LUC-*VvXTH10*, *VvEXPLA1* and *VvLBD13*) were transferred into *Agrobacterium tumefaciens* strain *GV3101*. The effector vector solution (2 mL) were blended with reporter vector solution (8 mL) and the commixture was incubated at 28°C and darkness for 3 h. The *Agrobacterium* bacterial solution was infiltrated using a 1-mL syringe into tobacco (*Nicotiana benthamiana*) leaves (~4 WAFB). Three days after infiltration, firefly luciferase (LUC) and *Renilla* luciferase (REN) activities were detected using a dual-luciferase reporter kit (DD1205-01, Vazyme, China). A multifunctional microplate reader (Spark, Tecan, Switzerland) was employed to measure LUC/REN ratios, which facilitated the evaluation of the transcriptional activities of VvARF19 on the *proVvLBD13* promoter and VvLBD13 on the promoters of *VvXTH10* and *VvEXPLA1*.

### Yeast one-hybrid and electromobility shift assays

The CDSs of *VvARF19* and *VvLBD13* from ‘Shine Muscat’ were linked to pB42AD, and the promoters of *VvEXPLA1* and *VvLBD13* (~2000 bp) from ‘Shine Muscat’ were cloned into the pLacZi and pAbAi vectors. In addition, the CDSs of *VvLBD13* from ‘Shine Muscat’ were linked to AD vector, and the promoter of *VvXTH10* from ‘Shine Muscat’ was cloned into pAbAi vector.

Primer sequences are shown in Supplementary Data Table S2. The plasmids of VvARF19-pB42AD and pro*VvLBD13-*pLacZi, pB42AD and pro *VvLBD13*-pLacZi, VvARF19-pB42AD and pLacZi, VvLBD13-pB42AD and pro*VvEXPLA1-*pLacZi, pB42AD and pro*VvEXPLA1*-pLacZi, VvLBD13-pB42AD, and pLacZi and pLacZi and pB42AD were together plated onto yeast strain EGY48 and plated on solid SD (without Ura and Trp). The resulting five colonies were resuspended in 5 μL water and dotted into SC medium (without Ura and Trp) with X-gal at 28°C for 4–24 h. AD-VvLBD13 and empty AD were transformed into the Y1H Gold yeast strain carried by pAbAi-pro*VvXTH10*. The yeast strain with AD-VvLBD13 and pAbAi-pro*VvXTH10* and AD and pAbAi-pro*VvXTH10* was grown on SD without Leu medium under 0, 50, 150, or 200 ng/mL ‌Aureobasidin A (AbA). Colonies were imaged using a Canon 60D.

The CDSs of *VvLBD13* and *VvARF19* were cloned into the pET-32a (+) vector using recombinant *VvLBD13* and *VvARF19* plasmids to generate His fusion protein with 2.0 mM of isopropyl-β-d-thiogalactopyramoside (IPTG) for 20 h at 16°C. Primer sequences are shown in Supplementary Data Table S2. Purification of VvLBD13 and VvARF19 fusion proteins was performed using His Sefinose resin (L00250/L00250-C, Genscript, China). Cold probes (not biotin-labeled at the 5′ end) and biotin-labeled probes were incubated at 25°C for 30 min using an EMSA kit (20 148; Thermo Fisher Scientific, Waltham, MA, USA). The sequences of the *VvLBD13*, *VvXTH10*, and *VvEXPLA1* promoter probes are shown in Supplementary Data Table S2.

### Genetic transformation of *VvARF19*, *VvLBD13*, and *VvXTH10* into grape berry and tomato plants

The full CDSs of *VvARF19*, *VvLBD13*, and *VvXTH10* were inserted into the 35S:SAK_277_ vector. The non-conservative *VvARF19*, *VvLBD13*, and *VvXTH10* fragments were cloned into TRV empty vector with the 35S promoter. Primer sequences are shown in Supplementary Data Table S2.

All of the recombined plasmids were introduced into *A. tumefaciens* GV3101 and then used for transient berry transformation. Fruit infiltrations were performed as described [54]. For transient injection, berry clusters with similar maturity in the field were firstly selected, and each grape cluster (80–100 berries) was divided into two parts: one half was used as the experimental group (injection of the target genes) and the other half as the control group (injection of the empty vector). *Agrobacterium* solution (200 μL) was injected into one berry using a 1-mL syringe during 7–8 WAFB. Samples were collected to measure the firmness, hemicellulose content, and gene expression level of grape berries.

Transgenic tomato (Micro-Tom) plants were procured via *Agrobacterium*-mediated transformation. The method used for generating transgenic plants of tomato was from Ye *et al*. [55]. Transgenic fruits from the *T*_1_ generation were collected to evaluate the firmness, hemicellulose content, and gene expression level of transgenic plants at immature stage (IM), breaker (Br) breaker + 3 days (Br + 3), breaker + 6 days (Br + 6), and breaker + 9 days (Br + 9).

### RT–PCR analysis of targeted genes in transgenic tomato and transiently transformed grape berries

Reverse transcription of total RNA separated from grape or tomato fruits was performed with Universal SYBR qPCR Master Mix (Vazyme, Q511-02). The program of SYBR qPCR Master Mix was 95°C for 5 min, followed by 40 cycles of 95°C for 10 s and 60°C for 30 s. Relative quantification of each gene expression level was done by the 2^(−ΔΔCt)^ method with the cycle threshold (Ct), with *GAPDH* used as the reference gene. The specific primers used for RT–PCR amplifications are shown in Supplementary Data Table S2.

### Statistical analysis

Difference analysis of all data was performed with Prism 8.0.1 software (GraphPad, San Diego, CA, USA). *P* > 0.05 was regarded as a non-significant difference.

## Supplementary Material

Web_Material_uhae322

## Data Availability

All data needed to support the conclusions in this paper are present in the paper and/or Supplementary Data Tables S1–4.
